# Pneumococcal Serotypes and Mortality following Invasive Pneumococcal Disease: A Population-Based Cohort Study

**DOI:** 10.1371/journal.pmed.1000081

**Published:** 2009-05-26

**Authors:** Zitta B. Harboe, Reimar W. Thomsen, Anders Riis, Palle Valentiner-Branth, Jens Jørgen Christensen, Lotte Lambertsen, Karen A. Krogfelt, Helle B. Konradsen, Thomas L. Benfield

**Affiliations:** 1 Department of Bacteriology, Mycology, and Parasitology, Statens Serum Institut, Copenhagen; 2 Department of Clinical Epidemiology, Aarhus University Hospital, Aalborg; 3 Department of Epidemiology, Statens Serum Institut, Copenhagen; 4 Department of Infectious Diseases and Clinical Research Centre, Hvidovre University Hospital, Copenhagen; 5 Faculty of Health Sciences, University of Copenhagen, Copenhagen; Emory University, United States of America

## Abstract

**Background:**

Pneumococcal disease is a leading cause of morbidity and mortality worldwide. The aim of this study was to investigate the association between specific pneumococcal serotypes and mortality from invasive pneumococcal disease (IPD).

**Methods and Findings:**

In a nationwide population-based cohort study of IPD in Denmark during 1977–2007, 30-d mortality associated with pneumococcal serotypes was examined by multivariate logistic regression analysis after controlling for potential confounders. A total of 18,858 IPD patients were included. Overall 30-d mortality was 18%, and 3% in children younger than age 5 y. Age, male sex, meningitis, high comorbidity level, alcoholism, and early decade of diagnosis were significantly associated with mortality. Among individuals aged 5 y and older, serotypes 31, 11A, 35F, 17F, 3, 16F, 19F, 15B, and 10A were associated with highly increased mortality as compared with serotype 1 (all: adjusted odds ratio ≥3, *p*<0.001). In children younger than 5 y, associations between serotypes and mortality were different than in adults but statistical precision was limited because of low overall childhood-related mortality.

**Conclusions:**

Specific pneumococcal serotypes strongly and independently affect IPD associated mortality.

## Abstract 2

Analyzing population-based data collected over 30 years in more than 18,000 patients with invasive pneumococcal infection, Zitta Harboe and colleagues find specific pneumococcal serotypes to be associated with increased mortality.

## Editors' Summary

**Background:**

Pneumococcal diseases—illnesses caused by *Streptococcus pneumoniae* bacteria—are leading causes of illness and death around the world. *S. pneumoniae* is transmitted through contact with infected respiratory secretions and usually causes noninvasive diseases such as ear infections and bronchitis. Sometimes, however, the bacteria invade the lungs (where they cause pneumonia), the bloodstream (where they cause bacteremia), or the covering of the brain (where they cause meningitis). These invasive pneumococcal diseases (IPDs) are often fatal. One million children die annually from pneumococcal disease, many of them in developing countries. In the developed world, however, IPDs mainly affect elderly people and patients with chronic conditions such as diabetes and alcoholism. Although pneumococcal diseases can sometimes be treated successfully with antibiotics, many patients die or develop long-term complications. Consequently, vaccination with “pneumococcal polysaccharide vaccine” (PPV) is recommended for everyone over 65 years old and for people between 2 and 65 years old who are at high risk of developing IPD; vaccination with “pneumococcal conjugate vaccine” (PCV) is recommended for children younger than 2 years old who are at high risk of IPDs.

**Why Was This Study Done?:**

*S. pneumoniae* is not a single organism. There are actually more than 90 *S. pneumoniae* variants or “serotypes.” These variants are coated with different polysaccharides (complex sugar molecules) that are, in part, responsible for the deleterious effects of *S. pneumonia* infections. The same molecules also trigger the human immune response that kills the bacteria. Consequently, pneumococcal vaccines contain polysaccharide mixtures isolated from the *S. pneumoniae* serotypes responsible for most pneumococcal disease. But are these serotypes also responsible for most of the deaths caused by IPD? Until now, the few studies that have investigated the association between *S. pneumoniae* serotypes and death from IPD have yielded conflicting results. Here, therefore, the researchers undertook a large population-based study to discover whether there is an association between specific pneumococcal serotypes and death following IPD.

**What Did the Researchers Do and Find?:**

The researchers linked data on the serotype of *S. pneumoniae* isolates sent to the Danish National Neisseria and Streptococcus Reference Center between 1977 and 2007 with clinical data from national medical databases. After allowing for other factors that might affect a person's likelihood of dying from IPD (for example, age and other illnesses), the researchers used multivariate logistic regression analysis (a statistical approach) to look for associations between *S. pneumoniae* serotypes and death within 30 days of admission to hospital for pneumococcal bacteremia or meningitis. Overall, 18% of the nearly 19,000 people included in this analysis died within 30 days of hospital admission; among the children younger than 5 years included in the study, the death rate was 3%. Among patients 5 years old or older, nine *S. pneumoniae* serotypes were associated with a more than 3-fold higher death rate (mostly from bacteremia) than serotype 1, the most common serotype isolated during the study. Interestingly, in young children, a different set of serotypes seemed to be associated with death. However, because so few children died from IPD, this result is statistically uncertain. The researchers' results also show that age, gender, having meningitis, having other illnesses, and alcoholism all affected a patient's chances of dying from IPD.

**What Do These Findings Mean?:**

These findings show that specific pneumococcal serotypes strongly affect the likelihood that a person aged 5 years or over will die within 30 days of admission to hospital with IPD. Importantly, unlike previous studies, this study was large and comprehensive—the Danish surveillance center covers more than 90% of the Danish population—and the researchers carefully took other factors into account that might have affected a patient's chances of dying from IPD. Thus, these new insights into which pneumococcal serotypes are most deadly could help in the design of new pneumococcal vaccines, at least for people aged 5 years or older. For younger children, however, the results are not as informative and a similar study now needs to be done in developing countries, where more young people die from IPD.

**Additional Information:**

Please access these Web sites via the online version of this summary at http://dx.doi.org/10.1371/journal.pmed.1000081.

- The US Centers for Disease Control and Prevention provides information for patients and health professionals on all aspects of pneumococcal disease and pneumococcal vaccination
- The US National Foundation for Infectious Diseases has a fact sheet on pneumococcal disease
- The UK Health Protection Agency also provides background information on pneumococcal disease
- The GAVI's Pneumococcal Vaccines Accelerated Development and Introduction Plan focuses on pneumococcal vaccines for children

## Introduction

Invasive pneumococcal disease (IPD) remains a major cause of morbidity and mortality worldwide despite the availability of antibiotic therapy and vaccines. Developing countries bear the major burden of IPD along with significantly higher rates of death and disability [1]–[3]. The introduction of a 7-valent pneumococcal conjugate vaccine (PCV7) for infants has significantly reduced rates of bacteremic and nonbacteremic pneumococcal disease in the United States and other industrialized countries, yet the emergence of nonvaccine pneumococcal serotypes may threaten the continued efficacy of conjugate vaccines [1],[4]–[6]. Unfortunately, since the prevalence of pneumococcal serotypes varies among geographical regions, the formulation of PCV7 is not optimal against the most prevalent serotypes found in the developing world [7]. In addition, some controversy surrounds the use of the available 23-valent polysaccharide vaccine (PPV23), which is recommended for patients of 2 y and older with high-risk conditions and for the elderly, since controlled trials were inconclusive and observational data suggested that PPV23 may prevent bacteremic but not nonbacteremic disease [8],[9].

Host and bacterial factors both contribute to IPD pathogenicity. Ethnicity, extremes of age, existence of comorbidities, alcoholism, and immunosuppression including HIV-1 infection are well-known risk factors associated with an increased susceptibility to IPD and are further associated with higher mortality [10]–[12]. The capsular polysaccharide is a major pneumococcal virulence factor that triggers a specific immunological response to infection and vaccination and constitutes an important determinant of the potential of *Streptococcus pneumoniae* to cause invasive and severe disease [13]–[15]. Experimental studies in animal models have shown that pneumococcal capsular serotypes differ in their ability to trigger inflammatory responses [16],[17]. Clinical studies in the pre- and postantibiotic era have also suggested that capsular polysaccharides are associated with specific manifestations of pneumococcal disease and mortality [10],[13],[15],[18]–[24]. However, only a few studies have evaluated the association of specific capsular serotypes with mortality from IPD in recent time and have delivered conflicting results [10],[20],[25],[26]. Most investigations were clinic-based studies from referral centers with few outcomes and limited confounder control, so their interpretation is limited.

Here we report our investigation of the association between specific invasive pneumococcal serotypes and mortality related to IPD, after controlling for important confounders, in a large population-based cohort study including more than 18,000 hospitalized IPD patients.

## Material and Methods

### Study Setting and Design

We conducted a nationwide population-based cohort study based on the linkage of IPD laboratory surveillance data to several Danish nationwide medical databases. Linkage between data sources was done by using the unique ten-digit identification number provided to all residents in Denmark [27]. The study was approved by the Danish Data Protection Agency (2007-41-0229).

Laboratory surveillance data on IPD were obtained from the National Neisseria and Streptococcus Reference Center (NSR), Statens Serum Institut (SSI), from 1 January 1977 through 31 December 2007. Isolates were routinely serotyped by pneumotest latex and Quellung reaction using type-specific pneumococcal rabbit antisera from SSI as previously described (SSI-Diagnostica, Copenhagen, Denmark) [28],[29]. Laboratory surveillance in Denmark is based on the voluntary submission of *S. pneumoniae* isolates cultured from a normally sterile site from all local departments of clinical microbiology to the NSR [30]. The coverage of the laboratory surveillance system has recently been estimated to reach more than 90% of the total Danish population [31].

PCV7 was introduced into the Danish childhood immunization program in October 2007 and the vaccine is currently administered free of charge to children at the ages of 3, 5, and 12 mo [31]. Between 2001 and 2007, vaccination with PCV7 was recommended only for children younger than 2 y belonging to high-risk groups [32]. An estimate of the proportion of the uptake of PCV7, based on the number of doses sold in Denmark (and assuming that all vaccines were given in a three-dose series to children younger than 2 y), ranges from 0.6 to 1.4 per 1,000 children between 2001 and 2006. The PPV23 has been recommended for patients older than 2 y at high risk and the elderly since 1996 in Denmark; however the uptake of PPV23 remains low. A similar estimate based on the number of doses of PPV23 sold per year (assuming that only persons ≥65 y, and older received one dose of the vaccine), suggest that the uptake has declined from approximately 30 to 17 doses per 1,000 persons aged 65 y and older between 1998 and 2007 (estimates based on unpublished data from SSI, Sales and Development Department, and The Danish Medicines Agency, http://www.dkma.dk/).

The percentage of invasive pneumococcal isolates with decreased susceptibility to β-lactam antibiotics or macrolides has remained low in Denmark, i.e., between 2% and 5% per year since 1991 when the first intermediate and resistant isolates were described [33],[34].

### Case Definition

A case was defined as an occurrence of IPD confirmed by positive culture for *S. pneumoniae* from a hospitalized patient's cerebrospinal fluid (CSF) or blood. When CSF isolates or both CSF and blood isolates were received simultaneously, the case was categorized as meningitis. Additionally, bacteremic patients who received a WHO International Classification of Disease (ICD)-8th or 10th code at discharge corresponding to bacterial meningitis (ICD-8 code 320, or ICD-10 codes A39, G00-G03) were considered to have a case of meningitis. All other cases were categorized as bacteremia. For patients with recurrent IPD episodes, only the first IPD episode was included in the analysis.

Only patients with IPD who where hospitalized within one week of the date of sampling were included in the study. Patients were excluded from the analysis due to one of the following reasons: (1) a provisional or invalid identification number (e.g., given to foreigners not residing in Denmark admitted to Danish hospitals), (2) emigration within 3 mo after the IPD admission, or (3) patients from whom samples were obtained outside the one-week criterion of admission to a hospital, as stated above.

### Data on Comorbidity and Outcome

All residents in Denmark had free access to primary care and hospitals provided by the National Health Services during the study period. The Danish National Hospital Register contains data on all hospitalizations since 1977 and on all outpatient visits since 1995 [35].

Comorbidity was assessed by computing the Charlson index score for each patient by using ICD codes from all available discharge diagnoses, as previously described [36],[37]. Briefly, the Charlson index is used to score patients' comorbid conditions in prognostic studies, and takes into account both the number and seriousness of comorbid diseases. We defined three levels of comorbidity prior to the IPD admission: low (score of 0), intermediate (1–2), and high (≥3).

Patients' discharge diagnoses were coded by physicians based on the ICD 8th revision (ICD-8, from 1977 through 1993) and the 10th revision (ICD-10) from January 1994. Since alcoholism-related conditions are not included in the Charlson index, we ascertained them separately by considering the following diagnosis codes: for ICD-8 classification: 291, 303, 979, 980, 577.10, 571.09, 571.10; and for ICD-10 classification: F10, G31.2, G62.1, G72.1, I42.6, K29.2, K86.0, K70, R78.0, T51, Z72.1.

The study's endpoint was death from any cause within 30 d following the IPD-related admission. Information on dates of death was obtained from the Danish Civil Registration System [27]. Data were retrieved 6 mo after the last patient was included in the study (1 July 2008).

### Statistical Analysis

Values for continuous variables are expressed as medians and interquartile ranges (IQRs). *p*-Values for the differences between groups were estimated by a Wilcoxon two-sample test. Associations between pneumococcal serotypes and 30-d mortality, after controlling for other confounders, were tested in multivariate analyses using a logistic regression model and expressed by odds ratio (OR) estimates with 95% confidence intervals (95% CIs). Crude and adjusted OR estimates for pneumococcal serotypes with a frequency of ≥50 cases were calculated. Cases of IPD caused by serotype 1 were chosen as the reference group, because serotype 1 represents the single most frequent serotype in our study population. The following variables were included in the logistic regression model: age, sex, IPD focus (bacteremia versus meningitis), comorbidity estimated by Charlson index, alcoholism-related conditions, and calendar period (1977–1986, 1987–1996, and 1997–2007). Depending on the explanatory model, IPD focus might or might not be considered an intermediate in the causal pathway between serotype and mortality, and analyses were therefore done both with and without inclusion of the focus of infection in the logistic regression model. Associations were tested separately among IPD patients younger than 5 y and those aged 5 y and older, and in the latter group further stratified by level of comorbidity (low, intermediate, high) and IPD focus (meningitis or bacteremia). *p*-Values<0.05 were considered significant. Data were analyzed using SAS Software, version 9.1 (SAS Institute, Cary, North Carolina, United States).

Serotypes were furthermore ranked according to their relative prevalence in the study population, by the crude number of deaths associated with each serotype, and by their population-attributable risk (PAR) of death among patients aged 5 y and older. The cumulative numbers of deaths associated with serotypes ranked in this manner were then compared with deaths associated with serotypes included in the existing PCV formulations containing up to 13 serotypes, using Chi-square tests with Yates correction, and two-tailed *p*-values were obtained. We calculated the PAR of death for each serotype using their prevalence proportion (PP) and adjusted mortality OR (as a measure of relative risk, RR) versus serotype 1 in the following equation [38]: PAR = PP×(RR−1)/(PP×[RR−1]+1), with 95% CIs computed as previously described [38],[39].The serotypes included in current and investigational pneumococcal protein conjugate vaccine formulations at the moment are: serotypes 4, 6B, 9V, 14, 18C, 19F, and 23F in the PCV7 (Wyeth); serotypes 1, 5, and 7F are additionally included in the ten-valent pneumococcal nontypeable *Haemophilus influenzae* protein D-conjugate vaccine (PHiD-CV; GlaxoSmithKline); and serotypes 3, 6A, and 19A in the 13-valent pneumococcal conjugate vaccine (PCV13; Wyeth).

## Results

### Study Population and Patient Characteristics

The Danish population numbered 5,080,000 inhabitants in 1977 and 5,447,084 in 2007. More than 90% of the population has a Danish ethnic background (http://www.statistikbanken.dk/). In total, 20,767 consecutive IPD cases were registered between 1 January 1977 and 31 December 2007. Of these, ∼10% (*n* = 1,970) were excluded from the present analysis for one of the following reasons: (1) patients who presented another focus than bacteremia or meningitis (*n* = 172), (2) patients who had no hospital admission recorded within 7 d before and after the sampling date (*n* = 1,782), and (3) emigration (*n* = 16). The serotype distribution and overall mortality among excluded patients was similar to that encountered in the study population (unpublished data).

In total, 18,858 patients with pneumococcal meningitis or bacteremia were included in the present study (Table 1). Approximately 90% of patients (*n* = 17,277) were 5 y and older, half were female (*n* = 9,424), and 15% had meningitis (*n* = 2,762). Among adolescents and adults (≥15 y), bacteremia patients were older than meningitis patients (median [IQR]: 68 y [54–78 y] versus 62 y [49–72 y]; *p*<0.001). Similarly, among children younger than 5 y, bacteremia patients were older than meningitis patients (1.2 y [0.9–1.8 y] versus 0.8 y [0.5–1.3 y], *p*<0.001) The overall 30-d mortality following the IPD-related admission was 18%, but 3% in children younger than 5 y, 14% in patients between 5 y and 64 y, and 24% among patients aged 65 y and older.

**Table 1:** Characteristics of patients hospitalized with IPD and 30-d mortality proportions by age group, Denmark, 1977–2007 (*n* = 18,858).

Category	Patients <5 y		Patients ≥5 y		Overall	
	Total	Mortality (%)	Total	Mortality (%)	Total	Mortality (%)
**Sex**						
Female	641	18 (3)	8,783	1,631 (18)	9,424	1,649 (17)
Male	940	23 (2)	8,494	1,730 (20)	9,434	1,753 (19)
**Charlson index**						
Low (0)	1,462	38 (3)	9,059	1,321 (14)	10,521	1,359 (13)
Intermediate (1–2)	108	1 (1)	5,536	1,247 (22)	5,644	1,248 (22)
High (≥3)	11	2 (18)	2,682	793 (30)	2,693	795 (30)
**Alcoholism-related conditions**						
No	1,581	41 (3)	16,131	3,019 (19)	17,712	3,060 (17)
Yes	—	—	1,146	342 (30)	1,146	342 (30)
**IPD focus**						
Bacteremia	1,067	17 (2)	15,029	2,776 (18)	16,096	2,793 (17)
Meningitis	514	24 (5)	2,248	585 (26)	2,762	609 (22)
**All**	1,581	41 (3)	17,277	3,361 (19)	18,858	3,402 (18)

### Serotype Distribution and Pneumococcal Serotype Crude Mortality Proportions

Capsular serotypes were available for all isolates obtained from IPD cases included in the study. We identified 77 different serotypes among invasive isolates including nonserotypeable isolates using pooled sera. No cases of serotypes 9L, 10C, 11F, 11C, 11D, 19C, 25A, 32A, 32F, 33D, 40, 47F, and 47A were identified.

Age-specific serotype distributions have previously been described in parts of the study period [31],[34]. Briefly, serotype 1 was the most common serotype and accounted for 15% of all IPD cases in the study population, followed by serotypes 14, 4, 7F, 9V, 3, 8, 12F, 23F, and 6B. Ten serotypes accounted for approximately 80% of IPD cases in children younger than 5 y: 14, 6B, 18C, 7F, 19F, 1, 6A, 23F, 4, and 9V. Serotypes 6B, 14, 7F, 18C, 19F, 6A, and 23F accounted for 70% of meningitis cases in this age group. Among patients aged 5 y and older, the most frequent serotypes were 1, 4, 14, 7F, 3, 9V, 12F, 8, and 23F, accounting for 67% of all cases. Serotype 3 was frequent across all age groups except children younger than 5 y, in whom it constituted less than 1% of the infections. Serotype 6B was less frequent among the elderly, while serotypes 4, 14, and 7F were found across all groups of patients.

**Figure 1:**
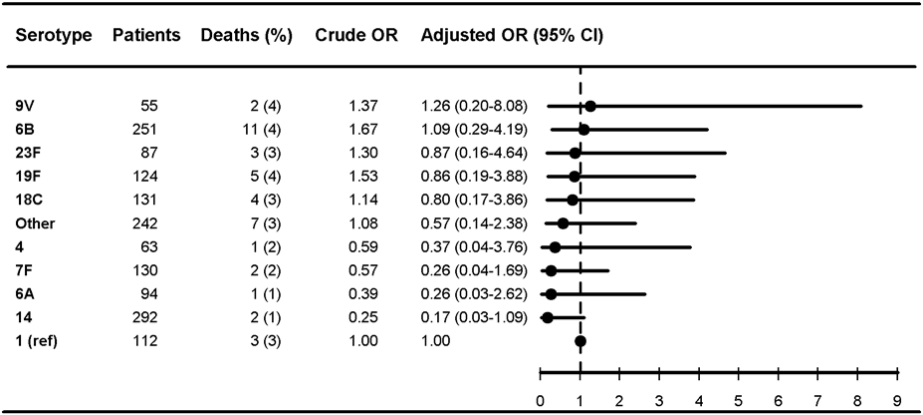
Multivariate logistic regression analysis of serotype-specific 30-d mortality associated with IPD in children younger than 5 y (*n* = 1,581). OR estimates adjusted for age (in years), sex, IPD focus (meningitis or bacteremia), time at diagnosis (in decades), alcoholism-related conditions, and low, medium, or high comorbidity score estimated by the Charlson index. The reference group was patients with IPD caused by serotype 1 in each group. ORs were calculated for serotypes with ≥50 IPD cases only.

Among meningitis cases, serotypes 7F, 14, 12F, 6B, 3, and 4 were each encountered in 6%–7% of patients. The serotype distribution in patients with different levels of comorbidity was similar across the strata, with serotypes 1, 3, 4, 8, 6B, 7F, 9V, 12F, 14, 19F, and 23F, causing more than 60% of IPD in patients with any level of comorbidity. Serotypes' specific prevalence and crude mortality proportions stratified by age, focus of infection, and level of comorbidity (only for patients aged 5 y and older) are shown in Figures 1–7.

**Figure 2 pmed-1000081-g002:**
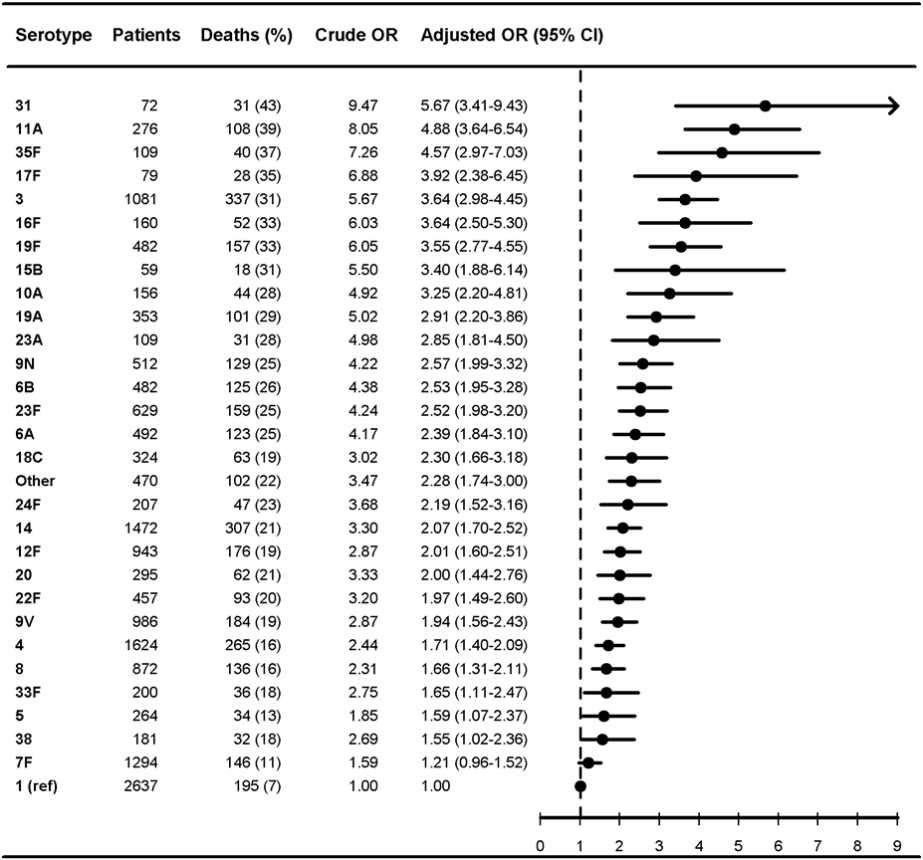
Multivariate logistic regression analysis of serotype-specific 30-d mortality associated with IPD in patients aged 5 y or older (*n* = 17,277). OR estimates adjusted for age (in years), sex, IPD focus (meningitis or bacteremia), time at diagnosis (in decades), alcoholism-related conditions, and low, medium, or high comorbidity score estimated by the Charlson index. The reference group was patients with IPD caused by serotype 1 in each group. ORs were calculated for serotypes with ≥50 IPD cases only.

**Figure 3 pmed-1000081-g003:**
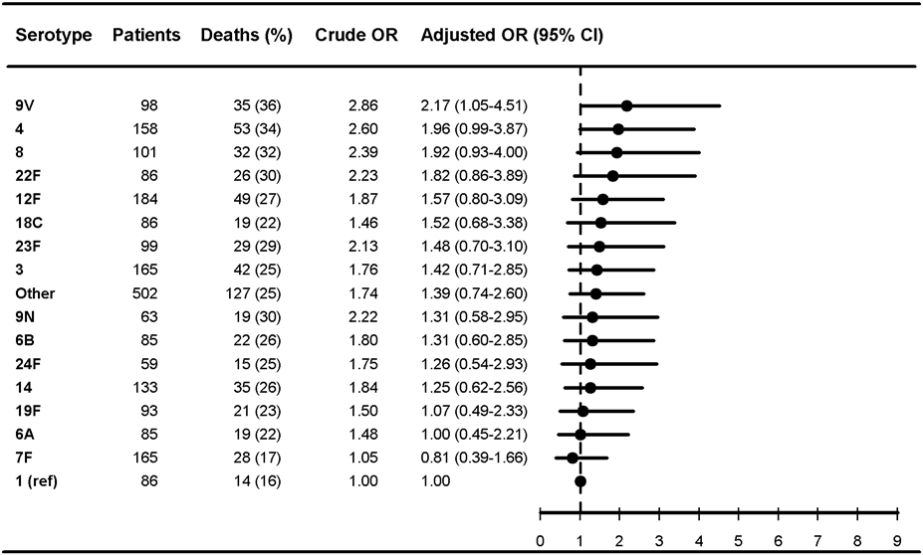
Multivariate logistic regression analysis of serotype-specific 30-d mortality associated with IPD in meningitis patients aged 5 y or older (*n* = 2,248). OR estimates adjusted for age (in years), sex, time at diagnosis (in decades), alcoholism-related conditions, and low, medium, or high comorbidity score estimated by the Charlson index. The reference group was patients with IPD caused by serotype 1 in each group. ORs were calculated for serotypes with ≥50 IPD cases only.

**Figure 4 pmed-1000081-g004:**
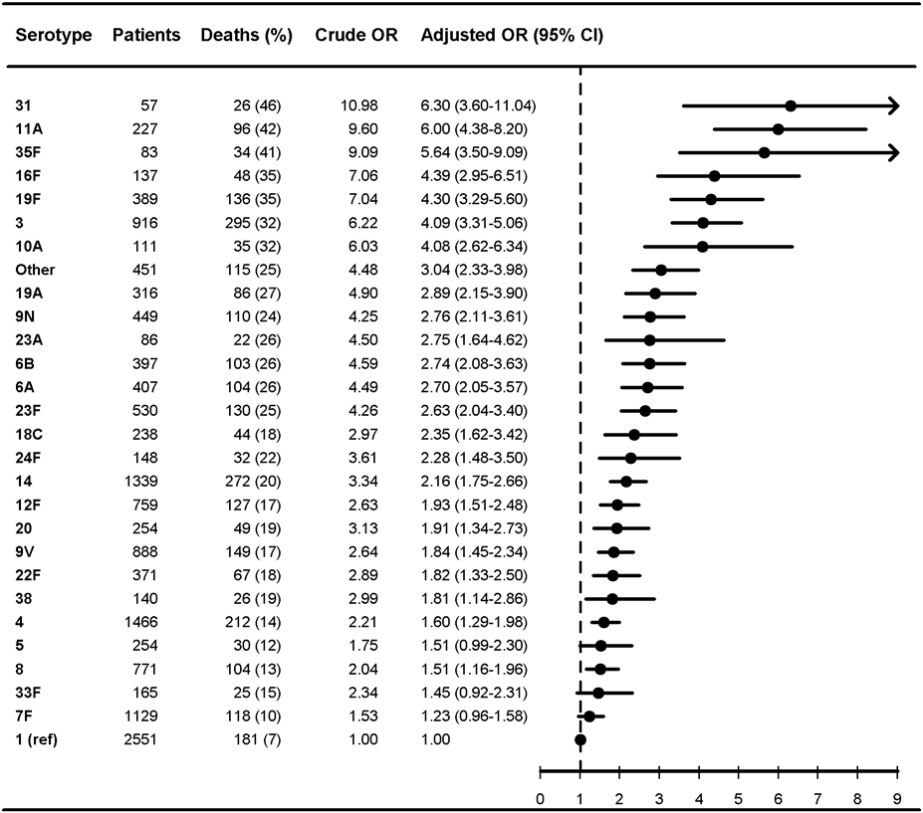
Multivariate logistic regression analysis of serotype-specific 30-d mortality associated with IPD in bacteremia patients aged 5 y or older (*n* = 15,029). OR estimates adjusted for age (in years), sex, time at diagnosis (in decades), alcoholism-related conditions, and low, medium, or high comorbidity score estimated by the Charlson index. The reference group was patients with IPD caused by serotype 1 in each group. ORs were calculated for serotypes with ≥50 IPD cases only.

**Figure 5 pmed-1000081-g005:**
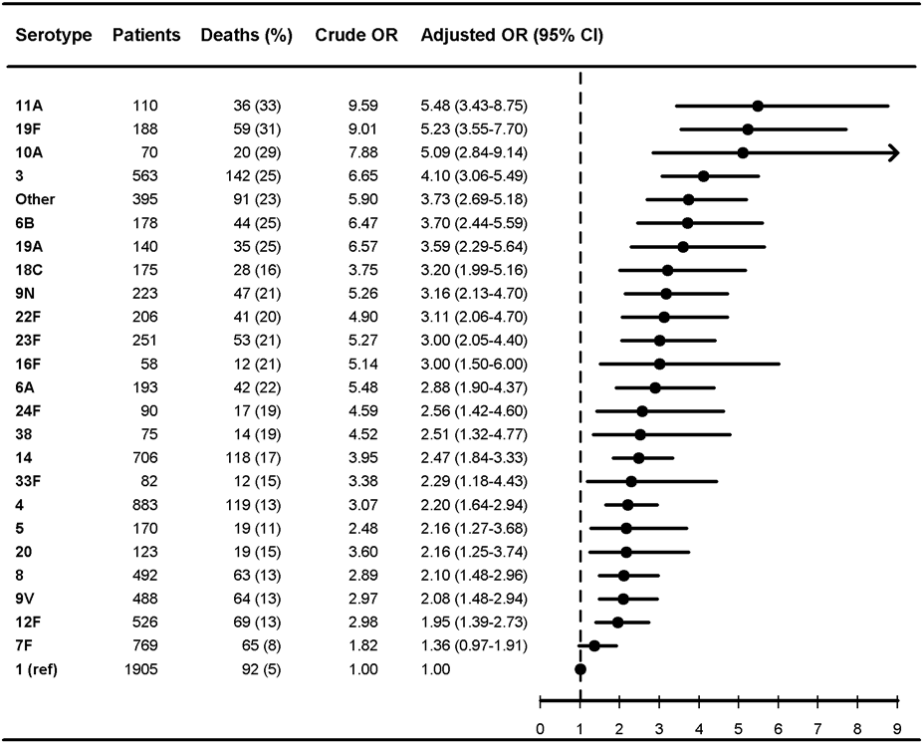
Multivariate logistic regression analysis of serotype-specific 30-d mortality associated with IPD in patients aged 5 y or older with low comorbidity level (Charlson 0) (*n* = 9,059). OR estimates controlled for age (in years), sex, IPD focus (meningitis or bacteremia), time at diagnosis (in decades), alcoholism-related conditions. The reference group was patients with IPD caused by serotype 1 in each group. ORs were calculated for serotypes with ≥50 IPD cases only.

**Figure 6 pmed-1000081-g006:**
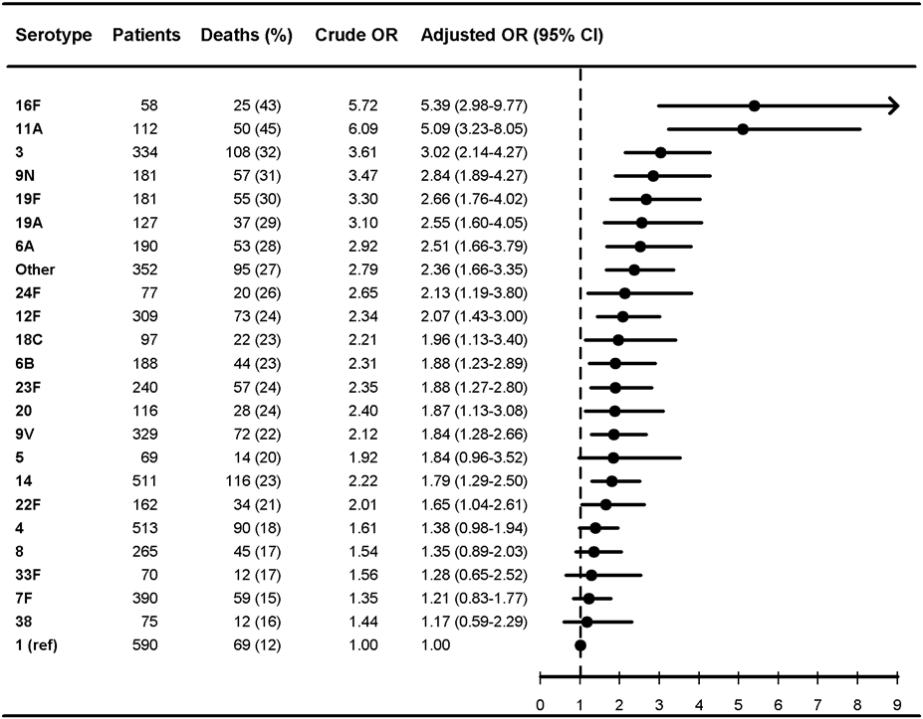
Multivariate logistic regression analysis of serotype-specific 30-d mortality associated with IPD in patients aged 5 y or older with intermediate comorbidity level (Charlson 1–2) (*n* = 5,536). OR estimates controlled for age (in years), sex, IPD focus (meningitis or bacteremia), time at diagnosis (in decades), alcoholism-related conditions. The reference group was patients with IPD caused by serotype 1 in each group. ORs were calculated for serotypes with ≥50 IPD cases only.

**Figure 7:**
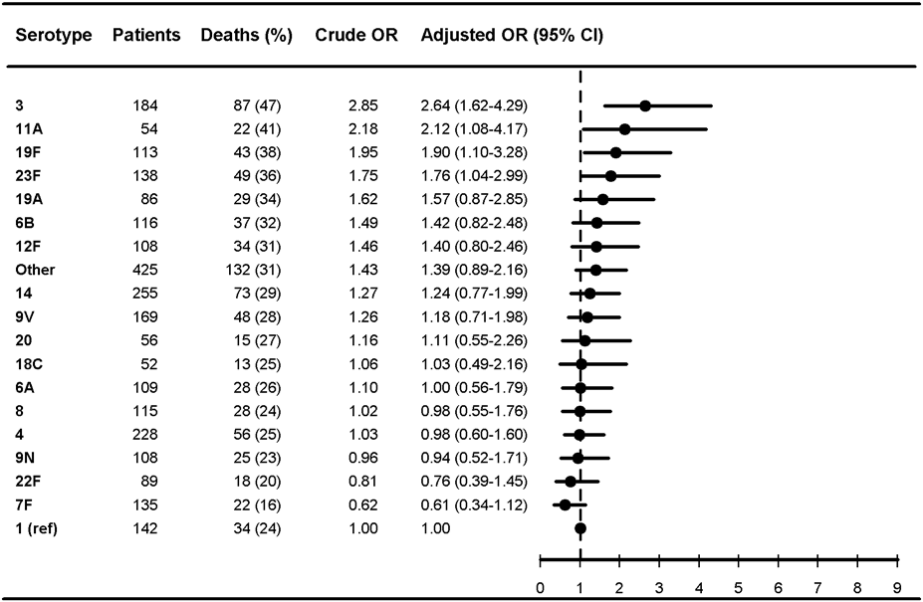
Multivariate logistic regression analysis of serotype-specific 30-d mortality associated with IPD in patients aged 5 y or older with high comorbidity level (Charlson 3+) (*n* = 2,682). OR estimates controlled for age (in years), sex, IPD focus (meningitis or bacteremia), time at diagnosis (in decades), alcoholism-related conditions. The reference group was patients with IPD caused by serotype 1 in each group. ORs were calculated for serotypes with ≥50 IPD cases only.

### Pneumococcal Serotypes and Adjusted 30-Day Mortality

Figures 1–7 show serotype-specific 30-d mortality proportions and crude and adjusted 30-d mortality OR estimates as compared with serotype 1. Results are shown for IPD patients stratified by age group: Figure 1 shows results for children younger than 5 y; Figure 2 shows results for patients 5 y or older. For patients aged 5 y and older the stratification was done also by IPD focus (Figures 3 and 4) and level of comorbidity (Figures 5–7). Only serotypes identified in ≥50 cases in every stratified model were included separately in the analysis. Serotypes identified in <50 cases were categorized as “other.”

After controlling for prognostic covariates, 27 serotypes were statistically significantly associated with increased 30-d mortality in the cohort of patients aged 5 y and older (Figure 2). Serotypes 31, 11A, 35F, 17F, 3, 16F, 19F, 15 B, and 10A were associated with adjusted OR estimates ≥3 as compared with serotype 1 in this age group. The associations were mainly driven by bacteremia cases (Figures 3 and 4). When not adjusting for IPD focus in the logistic regression model, the 30-d mortality OR estimates for most serotypes were even higher than for serotype 1, since focus adjustment controlled for the disposition of serotype 1 to cause bacteremia rather than meningitis, which has a worse prognosis (Tables S1 and S2).

Among children younger than 5 y, associations between serotypes and mortality tended to be different from those observed in older patients. In children younger than 5 y, the case fatality for serotype 1 was among the highest of the serotypes evaluated, while serotypes 14, 6A, 7F, and 4 tended to be associated with decreased mortality compared with serotype 1. However, because of low childhood mortality, statistical precision was limited and no statistically significant differences in mortality between serotypes were found (Figure 1).

Among patients aged 5 y and older with meningitis, most serotypes were associated with higher 30-d mortality than serotype 1, similar to findings in the bacteremia group, but we did not find statistically significant differences between serotypes (Figure 3). In patients aged 5 y and older with high levels of comorbidity, only serotypes 3, 11A, 19F, and 23F were found to be independently associated with mortality (Figure 7).

The following prognostic covariates included in the regression model together with pneumococcal serotypes were independently associated with 30-d mortality among patients aged 5 y and older: age per year increment (1.02 [1.02–1.03], *p*<0.001), male versus female sex (1.19 [1.09–1.29], *p*<0.001), meningitis versus bacteremia (1.90 [1.70–2.14], *p*<0.001), high Charlson index (1.85 [1.66–2.07], *p*<0.001) and intermediate Charlson index (1.35 [1.23–1.48], *p*<0.001) versus low Charlson index, a history of alcoholism-related conditions versus no such history (2.40 [2.08–2.78], *p*<0.001), and earlier decades of diagnosis: 1977–1986 (1.33 [1.16–1.52], *p*<0.001), and 1987–1996 (1.20 [1.10–1.31], *p*<0.001) compared to 1997–2007 (Table S2).

In children younger than 5 y, the following covariates were independently associated with 30-d mortality: meningitis versus bacteremia (2.05 [1.02–4.09], *p* = 0.04), high Charlson index (28.12 [4.34–181.83], *p*<0.001) versus low Charlson index, and earlier decades of diagnosis: 1977–1986 (3.28 [1.40–7.66], *p* = 0.006) compared to 1997–2007.

### Comparisons of Conjugate Vaccine Formulations Considering Prevalence and Mortality Data

To study whether alternative vaccine formulations that address serotype-specific mortality could prevent more cases and deaths, we used three approaches to rank serotypes: a prevalence-based score, a crude mortality score, and a PAR of death score, and compared them to current conjugate vaccine formulations. Because PARs of death were calculated based on confounder-adjusted mortality ORs, the resulting serotype ranking and cumulative number of deaths was different than that based on crude mortality. Due to the few deaths, low statistical precision, and many adjusted ORs below 1 versus the chosen reference group among children, we calculated PARs of death only among patients 5 y and older. Results are shown for children younger than 5 y in Table 2 and patients aged 5 y or older in Table 3.

**Table 2:** Cumulative number of cases and deaths in children younger than 5 y with IPD associated with the 13 serotypes included in current PCVs and 13 serotypes ranked according to their prevalence and associated crude number of deaths, Denmark, 1977–2007.

PCV Serotypes			Ranked by Prevalence			Ranked by Associated Crude Number of Deaths		
Serotype	Cumulative Cases	Cumulative Deaths	Serotype	Cumulative Cases	Cumulative Deaths	Serotype	Cumulative Cases	Cumulative Deaths
***14***	292	2	***14***	292	2	***6B***	251	11
***6B***	543	13	***6B***	543	13	***19F***	375	16
***18C***	674	17	***18C***	674	17	***18C***	506	20
***19F***	798	22	***7F***	804	19	***1***	618	23
***23F***	885	25	***19F***	928	24	***23F***	705	26
***4***	948	26	***1***	1,040	27	***14***	997	28
***9V***	1,003	28	***6A***	1,134	28	***7F***	1,127	30
***7F***	1,133	30	***23F***	1,221	31	***9V***	1,182	32
***1***	1,245	33	***4***	1,284	32	***9N***	1,197	34
***5***	1,256	33	***9V***	1,339	34	***6A***	1,291	35
***3***	1,269	34	***19A***	1,373	34	***4***	1,354	36
***6A***	1,363	35	***24F***	1,404	34	***3***	1,367	37
***19A***	1,397	35	***12F***	1,428	34	***15C***	1,378	38

There were no statistically significant differences (Chi-square test) between the cumulative number of deaths due to serotypes included in current PCV and the cumulative number of deaths due to serotypes ranked according to prevalence and crude number of deaths, respectively.

**Table 3:** Cumulative number of cases and deaths in patients aged 5 y and older with IPD associated with the 13 serotypes included in current PCVs and 13 serotypes ranked according to their prevalence, associated crude number of deaths, and the PAR of death, Denmark, 1977–2007.

PCV Serotypes			Ranked by Prevalence			Ranked by Associated Crude Number of Deaths			Ranked by PAR of Death			
Serotype	Cumulative Cases	Cumulative Deaths	Serotype	Cumulative Cases	Cumulative Deaths	Serotype	Cumulative Cases	Cumulative Deaths	Serotype	PAR (95%CI)	Cumulative Cases	Cumulative Deaths
***4***	1,624	265	***1***	2,637	195	***3***	1,081	337	***3***	14.2 (9.9–19.2)	1,081	337
***14***	3,096	572	***4***	4,261	460	***14***	2,553	644	***14***	8.35 (4.9–12.5)	2,553	644
***9V***	4,082	756	***14***	5,733	767	***4***	4,177	909	***19F***	6.63 (4.0–10.2)	3,035	801
***23F***	4,711	915	***7F***	7,027	913	***1***	6,814	1,104	***4***	6.25 (3.0–10.2)	4,659	1,066
***6B***	5,193	1,040	***3***	8,108	1,250	***9V***	7,800	1,288	***11A***	5.84 (3.3–9.5)	4,935	1,174
***19F***	5,675	1,197	***9V***	9,094	1,434	***12F***	8,743	1,464	***1***	5.80	7,572	1,369
***18C***	5,999	1,260	***12F***	10,037	1,610	***23F***	9,372	1,623	***23F***	5.23 (2.9–8.3)	8,201	1,528
***1***	8,636	1,455	***8***	10,909	1,746	***19F***	9,854	1,780	***12F***	5.21 (2.7–8.5)	9,144	1,704
***7F***	9,930	1,601	***23F***	11,538	1,905	***7F***	11,148	1,926	***9V***	5.11 (2.6–8.4)	10,130	1,888
***5***	10,194	1,635	***9N***	12,050	2,034	***8***	12,020	2,062	***9N***	4.45 (2.4–7.3)	10,642	2,017
***3***	11,275	1,972	***6A***	12,542	2,157	***9N***	12,532	2,191	***6B***	4.09 (2.1–6.8)	11,124	2,142
***6A***	11,767	2,095	***19F***	13,024	2,314	***6B***	13,014	2,316	***6A***	3.80 (1.9–6.4)	11,616	2,265
***19A***	12,120	2,196	***6B***	13,506	2,439	***6A***	13,506	2,439	***19A***	3.76 (1.9–6.4)	11,969	2,366

See text for calculation of PARs of death and respective 95% CIs. The PAR for serotype 1 (reference group) was estimated as the crude proportion of deaths caused by serotype 1 among all deaths in patients of 5 y and older (195 of 3,361 deaths). The cumulative number of deaths due to the first seven serotypes ranked according to prevalence, crude number of deaths, and PAR of death in patients aged 5 y and older was statistically significantly higher (Chi-square test) than the cumulative number of deaths due to serotypes included in the current PCV7 our population . For vaccines containing ten or more serotypes the differences in cumulative number of deaths were not statistically significant.

In children younger than 5 y, a vaccine containing either the most prevalent serotypes in our population, the serotypes with the highest associated crude mortality and the serotypes contained in current vaccines formulations (PCV7, PHiD-CV, and PCV13), would potentially prevent a comparable number of deaths.

In patients aged 5 y and older, an empirical vaccine containing either the seven most prevalent serotypes in our population, the seven serotypes with the highest associated crude mortality, or the highest PARs of death would potentially prevent a higher number of deaths compared with PCV7. However, for vaccines containing ten or more serotypes the differences were not statistically significant.

## Discussion

Our study is, to our knowledge, the largest and most comprehensive population-based study to date that evaluates short-term mortality associated with invasive pneumococcal serotypes after adjustment for possible confounders. The results support the hypothesis that specific pneumococcal capsular serotypes are significantly and independently associated with short-term mortality in patients with IPD. This observation is of particular interest in the era of conjugate vaccination, because a limited number of serotypes are included in the vaccines and because of the current emergence of nonvaccine serotypes.

Our results confirm that age, comorbid conditions, and clinical presentation affect serotype distribution as well as determine outcome from IPD [10],[20],[24],[40] (Figures 1–7). Few other population-based studies have investigated confounder-adjusted serotype-specific mortality, and they produced conflicting results and explored a relatively limited number of serotypes [10],[19],[21],[24],[25],[41],[42].

In accordance with our findings, previous studies have suggested that highly invasive serotypes, e.g., serotypes 1, 7F, and 14, are associated with lower mortality proportions and are more likely to be invasive in younger patients who have fewer comorbid conditions [16],[21],[41]. The pathogenic basis for these observations is unknown. Epidemiological and experimental studies have identified serotype 3 and 11A and serotypes included in serogroup 9 as serotypes that are more likely to cause severe disease [16],[19],[20],[41]. Future research should be directed at identifying virulence mechanisms that could improve our understanding of these associations and should explore potential implications for the clinical management of IPD patients.

In pneumococcal meningitis, experimental studies in animal models have indicated that there are serotype-related differences in the inflammatory response and in brain damage [16],[17]. Overall, we found fewer differences in mortality among all serotypes that caused meningitis, indicating that host factors are more important than serotypes as determinants of outcome from pneumococcal meningitis. Further clinical and experimental studies are needed in this field in order to elucidate whether differences in the early inflammatory response of the host lead to differences in severity of disease and short-term mortality after pneumococcal meningitis. Furthermore, studies exploring the notion that certain serotypes are more likely to cause meningitis than other less severe forms of pneumococcal disease should be conducted in the future.

Since the introduction of universal conjugate vaccination in several industrialized countries [43], the clinical epidemiology of IPD has changed dramatically, and “replacement” disease caused by nonvaccine serotypes has caused concern [5],[44]–[46]. Our results indicate that serotypes recently identified as potential (re-)emerging serotypes, such as serotype 3 and 19A, are associated with relatively high mortality in individuals aged 5 y and older compared with serotype 1 and other serotypes included in PCV7 [4],[5],[20]. Other serotypes (e.g., 31, 11A, 35F, 17F, 16F, 10A, 15B, 23A, and 9N) that have not yet been identified as emerging serotypes were also associated with higher mortality than serotype 1.

Our results support that comorbidity plays an important role in mortality after IPD [10],[21],[47]. Interestingly, a Swedish study [21] recently indicated that comorbidity may influence the invasiveness of specific serotypes in susceptible populations, suggesting that serotypes with low invasive disease potential may behave as opportunistic pathogens in patients with underlying comorbidity. We observed that patients with high levels of comorbidity were overrepresented among cases of serotypes 31, 11A, 35F, 17F. This observation supports the opportunistic pathogen hypothesis [21] and is of potential concern because serotype replacement may have particular implications for immunocompromised individuals, including HIV-1–infected individuals, in terms of severity of disease and related mortality.

In this study, pneumococcal serotypes other than those in older patients tended to cause the highest mortality estimates in children younger than 5 y, who are the primary target for conjugate pneumococcal vaccination. However, no statistically significant serotype–mortality associations could be demonstrated for children even in this large cohort, because of the very low overall childhood IPD-related mortality (less than 3%). Nonetheless, significant differences in serotype-specific mortality and severity of disease in children might be observed in developing countries, where childhood mortality ranges from 10% to 40% [1] due to, among many other factors, poorer access to health care and a higher prevalence of comorbid conditions, including HIV-1 and malnutrition [42].

The number of serotypes included in multivalent pneumococcal vaccines is limited. Therefore, we explored an alternative strategy to formulate a conjugate vaccine that took serotype-specific mortality into account. Our results indicate that current strategies for including serotypes in conjugate vaccines are appropriate for children and for adults, if the vaccine contains at least ten serotypes, in our study population. This is an interesting finding because trials with pneumococcal conjugate formulations are currently being conducted in adults and immunocompromised patients (http://www.clinicaltrials.gov/).

Overall, the results of this study indicate that there has been a decline in mortality after IPD during the last 30 y. This may be at least partly explained by the introduction of more sensitive diagnostic methods and improvements in therapy [48]. In spite of these advancements, mortality following IPD in Denmark remains remarkably high among adults, and changes in the trends in mortality for particular groups of patients over time deserve to be explored further in detail.

Our study has strengths and limitations. The large population we studied was well-defined, and the follow-up was complete because we used nationwide registries. Because there is universal health coverage in Denmark, we probably identified nearly all IPD episodes requiring hospitalization. We were able to adjust for a wide range of potential confounders through access to independent medical databases providing a complete medical history. Misclassification of data on comorbidity and other confounders might have led to some residual confounding, but we do not expect misclassification to be differentially related to serotypes. Moreover, we could not explore differences concerning other pneumococcal virulence factors (e.g., pneumolysin, hyaluronidase, catalase, and choline-binding protein A) or antibiotic resistance. Such virulence factors may also play a role in IPD prognosis and are a field for future research.

Our odds ratio estimates obtained by logistic regression somewhat overestimated the true relative risk of mortality associated with a given serotype if the mortality proportion was high. We evaluated different regression models, and logistic regression was the most robust and appropriate statistical model to evaluate the association between pneumococcal serotypes and 30-d mortality, since outcome was heterogeneous between serotypes and fatal events were rare in subgroups of patients, particularly among children. Even in this large study, it was not possible to fully explore the association between serotypes and mortality in all subgroups. For instance, we found that serotypes 3 and 19A were associated with high-mortality OR estimates in older children and adults compared to serotype 1, but we could not evaluate the strength of these associations in children younger than 5 y because of the low number of cases in this age group.

To our knowledge, this is the first and most comprehensive population-based study to date that shows an important role of individual pneumococcal capsular serotypes in the prognosis associated with IPD.

## Supporting Information

Alternative Language Abstract S1 **Translation of the abstract into Spanish by ZBH.**
(0.05 MB DOC)

Alternative Language Abstract S2 **Translation of the abstract into Danish by TLB.**
(0.03 MB DOC)

Table S1 **Serotype-specific 30-d mortality associated with invasive pneumococcal disease among patients aged 5 y and older.** Adjusted 30-d mortality ORs are shown without and with inclusion of IPD focus (bacteremia, meningitis) in the regression model.
(0.06 MB DOC)

Table S2 **Factors associated with 30-d mortality from invasive pneumococcal disease among patients aged 5 y and older.** Fully adjusted 30-d mortality ORs are shown without and with inclusion of IPD focus (bacteremia, meningitis) in the regression model.
(0.05 MB DOC)

## Abbreviations

CI: confidence interval
CSF: cerebrospinal fluid
ICD: International Classification of Disease
IPD: invasive pneumococcal disease
IQR: interquartile range
NSR: National Neisseria and Streptococcus Reference Center
OR: odds ratio
PAR: population-attributable risk
PCV7: 7-valent pneumococcal conjugate vaccine
PCV13: 13-valent pneumococcal conjugate vaccine
PHiD-CV: ten-valent pneumococcal nontypeable *Haemophilus influenzae* protein D-conjugate vaccine
PP: prevalence proportion
PPV23: 23-valent polysaccharide vaccine
RR: relative risk
SSI: Statens Serum Institut
