# Supplementary material for: Pneumococcal Serotypes and Mortality following Invasive Pneumococcal Disease: A Population-Based Cohort Study
Source: PLoS Med. 2009 May 26;6(5):e1000081. doi: 10.1371/journal.pmed.1000081 (PMC2680036; doi:10.1371/journal.pmed.1000081)
Supplement: Alternative Language Abstract S1 — Translation of the abstract into Spanish by ZBH. (0.05 MB DOC) [file pmed.1000081.s001.doc]

**Serotipos de neumococo y mortalidad relacionada con enfermedad neumocócica invasora: un estudio poblacional de cohortes**

Introducción: La enfermedad neumocóccica es una gran causa de morbilidad y mortalidad en todo el mundo. El objetivo de este estudio fue investigar la asociación entre los diversos tipos capsulares del neumococo (*S.pneumoniae*) y la mortalidad relacionada con la enfermedad neumocócica invasora.

Metodos y Resultados: Estudio retrospectivo de cohorte basado en los casos de enfermedad neumocócica invasora registrados en Dinamarca el periodo comprendido entre 1977-2007. La asociación entre los diversos serotipos del neumococo y la mortalidad a los 30 días posteriores al diagnóstico de enfermedad neumocócica invasora fueron evaluados usando el método de regresión logística ajustando por posibles factores de confusión. En total, 18.858 pacientes con enfermedad neumocócica invasora fueron incluídos en el estudio. La mortalidad durante los siguientes 30 días después de la enfermedad fue del 18% en el total de la cohorte y del 3% en niños menores de 5 años. Entre las variables incluídas en el modelo de regressión logística la edad, el sexo masculino, el diagnóstico de meningitis, altos niveles de comorbilidad, alcoholismo y temprana década en el diagnóstico se encontraron asociadas con la mortalidad de forma significativa. Entre los pacientes mayores de 5 años, los serotipos 31, 11A, 35F, 17F, 3, 16F, 19F, 15 B y 10A mostraron una estrecha asociación con la mortalidad tomando como comparador el serotipo 1 (todos con OR ajustadas ≥3, P <0.001). En pacientes menores de 5 años, las asociaciones entre serotipos y mortalidad fueron distintas pero de escasa precisión estadística debido a la baja mortalidad relacionada con la enfermedad neumocócica invasiva en este estudio.

Conclusión: Los serotipos capsulares del neumococo afectan de forma independiente la tasa de mortalidad relacionada con la enfermedad neumocócica invasora.
