# Supplementary material for: Pneumococcal Serotypes and Mortality following Invasive Pneumococcal Disease: A Population-Based Cohort Study
Source: PLoS Med. 2009 May 26;6(5):e1000081. doi: 10.1371/journal.pmed.1000081 (PMC2680036; doi:10.1371/journal.pmed.1000081)
Supplement: Alternative Language Abstract S2 — Translation of the abstract into Danish by TLB. (0.03 MB DOC) [file pmed.1000081.s002.doc]

**Pneumokok serotyper og dødelighed efter invasiv pneumokoksygdom: en befolkningsbaseret kohorte undersøgelse**

Baggrund: Pneumokoksygdom er en førende årsag til sygelighed og dødelighed i hele verden. Formålet med denne undersøgelse var at undersøge sammenhængen mellem specifikke pneumokok serotyper og dødelighed som følge af invasiv pneumokoksygdom (IPS).

Metoder og resultater: Landsdækkende kohorte undersøgelse af IPS i Danmark, 1977-2007. 30-dages dødelighed forbundet med pneumokok serotyper blev undersøgt med multivariat logistisk regressionsanalyse efter kontrol for mulige confounders. I alt 18.858 IPS patienter blev inkluderet. Samlet 30-dages dødelighed var 18% og 3% hos børn yngre end 5 år. Alder, mandligt køn, meningitis, høj komorbiditetsniveau, alkoholisme, og en diagnose i første 10-år af undersøgelsen var signifikant forbundet med øget dødelighed. Blandt personer 5 år og ældre var serotyperne 31, 11A, 35F, 17F, 3, 16F, 19F, 15 B og 10A forbundet med en stærkt øget dødelighed sammenlignet med serotype 1 (alle: justeret Odds ratio ≥ 3, P <0,001). Hos børn yngre end 5 år var sammenhængene mellem serotyper og dødelighed anderledes end hos voksne, men den statistiske præcision var begrænset på grund af den generelt lave IPS-relaterede børnedødelighed.

Konklusion: Specifikke pneumokok serotyper var uafhængigt forbundet med IPS dødelighed.
