# Supplementary material for: Pneumococcal Serotypes and Mortality following Invasive Pneumococcal Disease: A Population-Based Cohort Study
Source: PLoS Med. 2009 May 26;6(5):e1000081. doi: 10.1371/journal.pmed.1000081 (PMC2680036; doi:10.1371/journal.pmed.1000081)
Supplement: Table S1 — Serotype-specific 30-d mortality associated with invasive pneumococcal disease among patients aged 5 y and older. Adjusted 30-d mortality ORs are shown without and with inclusion of IPD focus (bacteremia, meningitis) in the regression model. (0.06 MB DOC) [file pmed.1000081.s003.doc]

**Supporting information Table S1: Serotype-specific 30-day mortality associated with invasive pneumococcal disease among patients of 5 years and older. Adjusted 30-day mortality ORs are shown without and with inclusion of IPD-focus (bacteremia, meningitis) in the regression model.**

| ***Without adjusting for IPD-focus*** | ***Adjusted OR (95% CI)*** | ***Adjusting for IPD-focus*** | ***Adjusted OR (95% CI)*** |
| --- | --- | --- | --- |
| ***31*** | 6.39 (3.84-10.63) | ***31*** | 5.67 (3.41 -9.43) |
| ***11A*** | 5.48 (4.10-7.33) | ***11A*** | 4.88 (3.64-6.54) |
| ***35F*** | 5.30 (3.45-8.14) | ***35F*** | 4.57 (2.97-7.03) |
| ***17F*** | 4.79 (2.92-7.86) | ***17F*** | 3.92 (2.38-6.45) |
| ***15B*** | 4.07 (2.21-7.33) | ***3*** | 3.64 (2.98-4.45) |
| ***19F*** | 4.04 (3.15-5.17) | ***16F*** | 3.64 (2.59-5.30) |
| ***16F*** | 4.02 (2.77-5.84) | ***19F*** | 3.55 (2.77-4.55) |
| ***3*** | 3.99 (3.22-4.87) | ***15B*** | 3.40 (1.88-6.14) |
| ***10A*** | 3.84 (2.60-5.67) | ***10A*** | 3.25 (2.20-4.81) |
| ***23A*** | 3.32 (2.11-5.21) | ***19A*** | 2.91 (2.20-3.86) |
| ***19A*** | 3.14 (2.38-4.16) | ***23A*** | 2.85 (1.81-4.50) |
| ***6B*** | 2.87 (2.22-3.71) | ***9N*** | 2.57 (1.99-3.32) |
| ***9N*** | 2.84 (2.20-3.66) | ***6B*** | 2.53 (1.95-3.28) |
| ***23F*** | 2.82 (2.22-3.57) | ***23F*** | 2.52 (1.98-3.20) |
| ***6A*** | 2.70 (2.09-3.50) | ***6A*** | 2.39 (1.84-3.10) |
| ***18C*** | 2.67 (1.93-3.69) | ***18C*** | 2.30 (1.66-3.18) |
| ***Other*** | 2.64 (2.01-3.46) | ***Other*** | 2.28 (1.74-3.00) |
| ***24F*** | 2.63 (1.83-3.79) | ***24F*** | 2.19 (1.52-3.16) |
| ***12F*** | 2.26 (1.81-2.83) | ***14*** | 2.07 (1.70-2.52) |
| ***22F*** | 2.24 (1.70-2.96) | ***12F*** | 2.01 (1.60-2.51) |
| ***14*** | 2.22 (1.82-2.71) | ***20*** | 2.00 (1.44-2.76) |
| ***20*** | 2.21 (1.60-3.05) | ***22F*** | 1.97 (1.49-2.60) |
| ***9V*** | 2.09 (1.68-2.61) | ***9V*** | 1.94 (1.56-2.43) |
| ***33F*** | 1.87 (1.25-2.78) | ***4*** | 1.71 (1.40-2.09) |
| ***4*** | 1.83 (1.49-2.23) | ***8*** | 1.66 (1.31-2.11) |
| ***38*** | 1.81 (1.19-2.74) | ***33F*** | 1.65 (1.11-2.47) |
| ***8*** | 1.79 (1.41-2.27) | ***5*** | 1.59 (1.07-2.37) |
| ***5*** | 1.61 (1.08-2.39) | ***38*** | 1.55 (1.02-2.36) |
| ***7F*** | 1.31 (1.04-1.65) | ***7F*** | 1.21 (0.96-1.52) |
| ***1*** | 1 | ***1*** | 1 |

*ORs adjusted for age (in years), sex, time at diagnosis (in decades), alcoholism-related conditions, and low, medium, or high comorbidity score estimated by the Charlson index. The reference group was patients with IPD caused by serotype 1 in each group. ORs were calculated for serotypes with ≥ 50 IPD-cases only
