# Supplementary material for: Pneumococcal Serotypes and Mortality following Invasive Pneumococcal Disease: A Population-Based Cohort Study
Source: PLoS Med. 2009 May 26;6(5):e1000081. doi: 10.1371/journal.pmed.1000081 (PMC2680036; doi:10.1371/journal.pmed.1000081)
Supplement: Table S2 — Factors associated with 30-d mortality from invasive pneumococcal disease among patients aged 5 y and older. Fully adjusted 30-d mortality ORs are shown without and with inclusion of IPD focus (bacteremia, meningitis) in the regression model. (0.05 MB DOC) [file pmed.1000081.s004.doc]

**Supporting information Table S2: Factors associated with 30-day mortality from invasive pneumococcal disease among patients of 5 years and older. Fully adjusted 30-day mortality ORs are shown without and with inclusion of IPD focus (bacteremia, meningitis) in the regression model.**

| ***Without adjusting for IPD-focus*** | ***Adjusted OR***  ***(95% CI)*** | ***Adjusting for IPD-focus*** | ***Adjusted OR (95% CI)*** |
| --- | --- | --- | --- |
| ***Age (per year)*** | 1.02 (1.02-1.03) | ***Age (per year)*** | 1.02 (1.02-1.03) |
| ***Sex*** |  | ***Sex*** |  |
| ***Female*** | 1 | ***Female*** | 1 |
| ***Male*** | 1.18 (1.09-1.28) | ***Male*** | 1.19 (1.09-1.29) |
| ***Comorbidity*** |  | ***Comorbidity*** |  |
| ***Charlson 0*** | 1 | ***Charlson 0*** | 1 |
| ***Charlson 1-2*** | 1.25 (1.14-1.37) | ***Charlson 1-2*** | 1.35 (1.23-1.48) |
| ***Charlson 3*** | 1.68 (1.50-1.87) | ***Charlson 3*** | 1.85 (1.66-2.07) |
| ***Calendar period*** |  | ***Calendar period*** |  |
| ***1997-2007*** | 1 | ***1997-2007*** | 1 |
| ***1987-1996*** | 1.22 (1.12-1.32) | ***1987-1996*** | 1.20 (1.10-1.31) |
| ***1977-1986*** | 1.43 (1.25-1.63) | ***1977-1986*** | 1.33 (1.16-1.52) |
|  |  |  |  |
| ***Alcoholism-related conditions*** |  | ***Alcoholism-related conditions*** |  |
| ***No*** | 1 | ***No*** | 1 |
| ***Yes*** | 2.29 (1.98-2.65) | ***Yes*** | 2.40 (2.08-2.78) |
|  |  | ***IPD-focus*** |  |
|  |  | ***Bacteremia*** | 1 |
|  |  | ***Meningitis*** | 1.91 (1.70-2.14) |

*ORs adjusted for age (in years), sex, time at diagnosis (in decades), alcoholism-related conditions, and low, medium, or high comorbidity score estimated by the Charlson index. Pneumococcal serotypes are included in the model. The reference group was patients with IPD caused by serotype 1 in each group. ORs were calculated for serotypes with ≥ 50 IPD-cases only
